# Chemical and Ultrastructural Characterization of Dentin Treated with Remineralizing Dentifrices

**DOI:** 10.3390/jfb15010025

**Published:** 2024-01-16

**Authors:** Dimitra Athanasiadou, Denise Eymael, Beshr Hajhamid, Karina M. M. Carneiro, Anuradha Prakki

**Affiliations:** 1 Dental Research Institute, Faculty of Dentistry, University of Toronto, Toronto, ON M5G 1G6, Canada; dimath@chalmers.se (D.A.); denise.lopeseymael@utoronto.ca (D.E.); beshr.hajhamid@utoronto.ca (B.H.); 2 Institute of Biomedical Engineering, University of Toronto, Toronto, ON M5S 3G9, Canada

**Keywords:** dentin, brushing, dentifrice, atomic force microscopy, electron microscopy, teeth

## Abstract

The aim of this study is to investigate dentin chemical and ultrastructural changes upon exposure to remineralizing dentifrices. Dentin disks were obtained from permanent human molars and treated for 7 days with the dentifrices: (1) C group—control (no dentifrice); (2) S group—Sensodyne Repair & Protect; (3) D group—Dentalclean Daily Regenerating Gel; and (4) DB group—D group + Dentalclean regenerating booster. Afterwards, samples were submitted to an additional 7 days of toothbrushing associated with daily acidic challenge. Samples were imaged and analyzed (days 1, 7, and 14) for Young’s modulus by atomic force microscopy (AFM), scanning electron microscopy (SEM) and energy-dispersive X-ray spectroscopy (EDX), transmission electron microscopy (TEM) and selected area electron diffraction (SAED). SEM and AFM revealed precipitate deposition on dentin surfaces in groups S, D, and DB, formed as early as day 1. Surface elemental analysis showed a Si increase on all brushed surfaces. Similar surface morphology was maintained after the acidic challenge period. Bright-field TEM/SAED revealed the formation of nanocrystalline hydroxyapatite inside the dentin tubules of groups S, D, and DB after day 7. Group C presented a gradual reduction of Young’s modulus from days-1–14, whereas all remaining groups had increased values. All evaluated dentifrices led to successful formation of hydroxyapatite and increased dentin stiffness.

## 1. Introduction

Dentin hypersensitivity is a common condition, with 25–46% prevalence among people between 18 and 70 years old [1,2]. The most accepted explanation for the mechanism of dentin hypersensitivity is the hydrodynamic theory. This theory suggests that a pain-provoking stimulus increases or alters the direction of the flow of dentinal fluid within the tubules, stimulating the nerves around odontoblast cells [1,3]. Two common strategies to treat dentin hypersensitivity are the closure of the dentinal tubules, to reduce fluid flow and dentin permeability, and the reduction of nerve excitability through specific chemicals [1,4,5].

There are several professional and home routine products to manage dentin hypersensitivity. However, it is still necessary to find a gold standard agent that presents a long-term effect in the management of hypersensitivity [1,4,6]. Mouthwashes, gels and dentifrices are the most used desensitizing agents because of their easy access and relatively low cost. These agents usually depolarize nervous impulses or occlude dentinal tubules [7,8,9]. Of these, there is a desensitizing dentifrice that is available on the market with the trade name of Sensodyne Repair & Protect (GlaxoSmithKline, Mississauga, ON, Canada), which is composed of calcium sodium phosphosilicate, a bioactive glass (NovaMin^®^ technology). This product undergoes several chemical reactions in aqueous solution, forming carbonated hydroxyapatite over the dentinal surface and therefore promoting remineralization [1,2,10,11,12].

An alternative dentifrice for dentin remineralization is Dentalclean Daily Regenerating Gel (Dentalclean, Londrina, PR, Brazil), which, according to the same manufacturer, can be associated with a regenerating booster component (Dentalclean, Londrina, PR, Brazil) (REFIX^®^ technology). The latter material uses the technology of calcium nanoparticles associated with phosphate salts, serving as a carrier for calcium to permeate dental tissues that may increase the efficacy of the remineralizing agents [13]. Both systems are capable of enhancing the action of 1450 ppm sodium fluoride compared with other commercial toothpastes [14,15]. Such a property would provide the sealing of dentinal tubules, as well as remineralization and inhibition of dentin demineralization [16]. However, studies on these products are scarce in the literature. No previous studies have used specific analytical methods to characterize the composition of toothpaste deposits, supporting the aim of the present in vitro study of chemically and ultrastructurally characterizing dentin properties upon exposure to remineralizing dentifrices. The null hypotheses tested are: (a) There will be no surface property differences between dentifrice-treated and untreated dentin. (b) An acidic challenge will not negatively affect the surface properties of dentin treated with remineralizing dentifrices.

## 2. Materials and Methods

### 2.1. Sample Preparation

**Figure 1:**
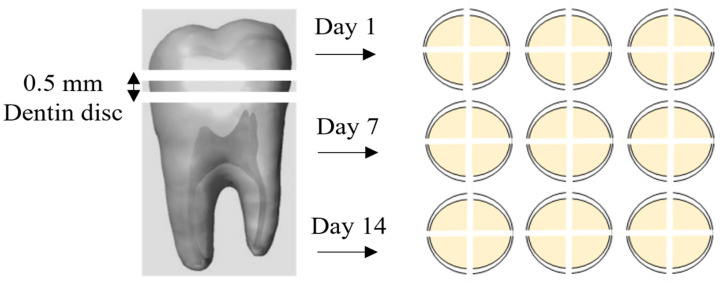
Schematic representation of study groups’ distributions.

Nine freshly extracted sound human third molars were collected and stored according to the local research ethics protocol (approved protocol #00038325). Teeth were cleaned with curettes and sterilized by gamma radiation at a dosage of 2.5 MRad [17]. Teeth with clinical evidence of caries, root resorption, cracks or fractures were not considered for the study. After cleaning, 0.5 mm-thick dentin discs were obtained from the teeth using a low-speed diamond saw (Isomet, Buehler Ltd., Lake Bluff, IL, USA) under water cooling. Wet silicon carbide 600-grit sandpaper was used for five seconds on the occlusal surface of each disc to standardize the smear layer. Next, two crossed scratches were made on the occlusal surface of each disc to divide it into four equal parts. Samples were equally and randomly distributed among the four study groups (*n* = 3) according to the type of dentifrice and testing timepoint (day 1, day 7 and day-14) (Figure 1). Dentifrices were used as follows: C group—control (artificial saliva; no dentifrice); S group—Sensodyne Repair & Protect; D group—Dentalclean Daily Regenerating Gel; and DB group—D group + Dentalclean regenerating booster (1:1 weight). A description of the composition of the products used in each group is shown in Table 1.

### 2.2. Simulated Toothbrushing

Samples were subjected to 14 consecutive days of toothbrushing followed by agitation in artificial saliva [18]. Soft bristle toothbrushes (Oral-B Healthy Clean, Soft, Procter & Gamble, Toronto, ON, Canada) were manually used by a single calibrated operator, who brushed each dentin quarter for twice a day in a uniform way (~2N, 45 strokes per 2 min) [19] after immersing the toothbrush in 10 mL of the dentifrice slurry. Dentifrice slurries were prepared right before using, by mixing one part of dentifrice with three parts (weight) of fluoride-free artificial saliva. Dentin discs were chemically and structurally analyzed after 1, 7 and 14 days of consecutive toothbrushing.

### 2.3. Acidic Challenge

From the period of 7 to 14 days of toothbrushing, the samples were also subjected to an acidic challenge. Specifically, the dentin discs were submerged in 0.5% citric acid solution (pH 2.5) [20] under agitation for two minutes and rinsed with distilled water for 30 s. The acidic challenge was performed daily, 1 h prior to the first toothbrushing.

### 2.4. Scanning Electron Microscopy (SEM) and Energy-Dispersive X-ray Spectroscopy (EDX)

Dentin discs were mounted on aluminum stubs and coated with a 2 nm-Pt layer using a Fisons Polaron SC515 sputter coater (LADD Research Industries, VT, USA). The microstructure and chemical composition of the dentin samples were examined using FEI Quanta FEG 250 SEM (ELECMI, Madrid, ES) operating in high-vacuum mode at 5 kV coupled with an energy-dispersive X-ray detector (EDX, Zeiss Supra V50, Carl Zeiss, Oberkochen, Germany). EDX was used for the quantitative elemental analysis of dentin precipitates. Three analytical points were selected for each sample (300 µm^2^) and the mean weight and atomic percent values of each detected element was calculated.

### 2.5. Atomic Force Microscopy (AFM)

A Multimode III AFM (Bruker, Santa Barbara, CA, USA) was used to image the air-dried surfaces of dentin samples and a JPK Nanowizard 4 AFM (JPK Instruments Ltd., Berlin, Germany) was used for Young’s modulus measurements. Commercially available cantilevers were used for the imaging (OTESPA-R3 probes, Bruker, spring constant 26 N/m) and for the force (RTESPA-525 probes, Bruker, spring constant 200 N/m) measurements. Force measurements (100 force curves) were performed in a 10 × 10 μm area per sample in triplicate. An optical microscope was combined with the AFM to control tip to sample positioning. Images were recorded using tapping mode and force curves were taken with force spectroscopy mode using the JPK NanoWizard^®^ software version 6.1.111 with a scan rate of 1 Hz to record the surface Young’s modulus values. Image processing was performed using NanoScope Analysis software (Bruker Corporation, Santa Barbara, CA, USA) and force curve analysis was performed using the JPK NanoWizard^®^ Analysis software version 6.1.111 (JPK Instruments, Berlin, Germany). The mean Young’s modulus values were subjected to 2-way ANOVA (toothpaste × timepoint) followed by a Tukey test (α = 0.05).

### 2.6. Transmission Electron Microscopy (TEM) and Selected-Area Electron Diffraction (SAED)

TEM analysis was performed on samples at day 7 to examine the crystallinity and mineral phase of newly formed material in the dentin. Treated dentin discs were fixed with 4% paraformaldehyde and 1% glutaraldehyde in 0.1 M phosphate buffer overnight at 4 °C, followed by dehydration and embedding in resin blocks. Resin blocks were cut using a Reichert Ultracut E ultra-microtome (Leica, Concord, ON, Canada). Bright-field TEM images of dentin cross sections and selected-area electron diffraction (SAED) patterns were acquired using a T20 Thermo TEM instrument operating at 120 kV. SAED patterns were recorded using an aperture that selects an area with a 200 nm diameter. Indexing of SAED patterns was performed by measuring the d-spacing using ImageJ software (version 1.8).

## 3. Results

### 3.1. Dentin Characterization and Chemical Composition

**Figure 2:**
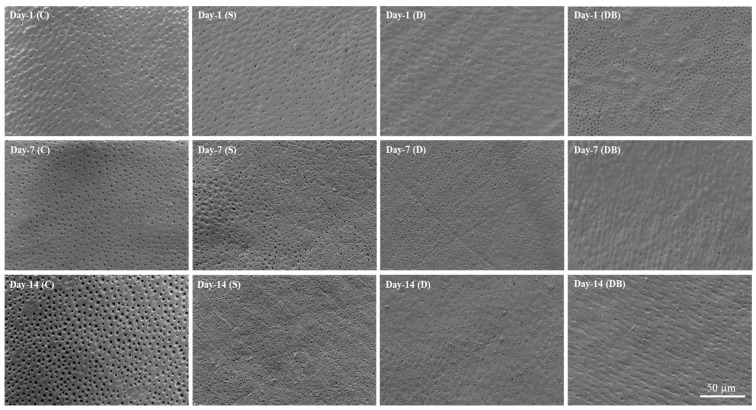
SEM images (2000× magnification) of evaluated dentin groups. Group C = control; group S = Sensodyne Repair & Protect; group D = Dentalclean Daily Regenerating Gel; group DB = group D + Dentalclean regenerating booster.

SEM imaging and EDX analysis revealed differences on the structure and chemical composition among the tested dentin samples. Figure 2 shows representative SEM images of treated dentin surfaces. Open dentin tubules can be observed on the group C images, which became more pronounced after the 14-day timepoint. After the first day of brushing, evident precipitate deposition compared with control was observed on the dentin surfaces of groups S, D and DB, whereby partially occluded tubules could be observed with sites of full coverage. For the groups S, D and DB, similar surface morphology was maintained after the 7-day and 14-day toothbrushing timepoints. The elemental mean percentage values of days 1, 7 and 14 are shown in Table 2, Table 3 and Table 4, respectively. Surface elemental analysis showed elemental variations among groups and timepoints, indicating modification of the dentin structure after toothbrushing. Elements of interest such as Si, Ca and P were identified on the mineralized layer. An interesting increase in Si was observed on all brushed surfaces for all toothpastes and timepoints (ranging from 4.0 to 12.7 weight%) in relation to the control mean values (ranging from 0.2 to 0.7 weight%).

**Figure 3:**
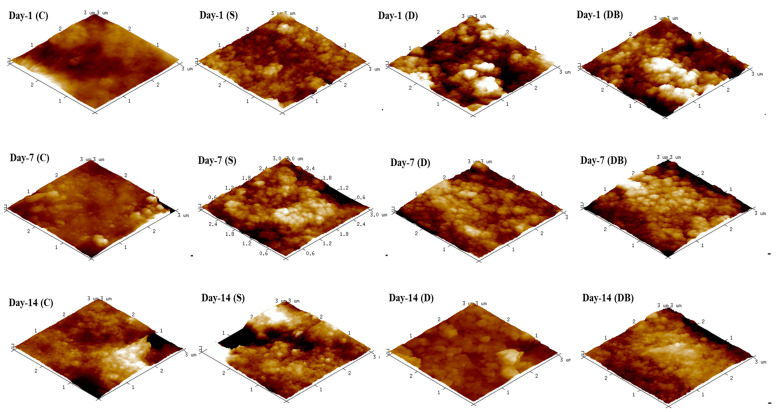
AFM images (3 × 3 µm = 9 µm^2^) of evaluated dentin groups. Group C = control; group S = Sensodyne Repair & Protect; group D = Dentalclean Daily Regenerating Gel; group DB = group D + Dentalclean regenerating booster.

AFM analysis shows the surface morphological changes for each group tested (Figure 3). It can be observed that group C shows a smoother surface, especially at the day-1 and day-7 timepoints. After the acidic challenge, group C (day 14) appeared rougher. As for the brushed samples, AFM imaging revealed a uniform distribution of globular structures throughout dentin surfaces that was visible for all toothpastes and at all evaluated timepoints.

### 3.2. Mechanical Properties of Remineralizing Dentin

The mean values for Young’s modulus are shown in Table 5. For Young’s modulus, two-way ANOVA revealed the significant effect of each factor, toothpaste (*p* = 0.00) and time (*p* = 0.00), as well as a significant interaction between factors (*p* = 0.00). A multiple comparison test showed a gradual decrease in Young’s modulus from day 1 to day 14 for group C. Conversely, an increase in Young’s modulus (day 1 to day 14) was noted for all the remaining groups (*p* > 0.05). Of these, group D showed statistical differences between day 1 and day 7 as well as between day 7 and day 14 (*p* < 0.01). Within each timepoint, on day 1 there was no significant difference among groups (*p* = 0.69). On day 7, group C values were statistically lower than groups S and D, and groups S and D were statistically lower than group DB. There was no difference between groups S and D (*p* = 0.70). On day 14, group C was statistically lower than group S (*p* < 0.01). Groups C and S were statistically lower than groups D and DB, which did not differ from each other (*p* = 0.43).

### 3.3. Transmission Electron Microscopy (TEM) and Selected-Area Electron Diffraction (SAED)

**Figure 4:**
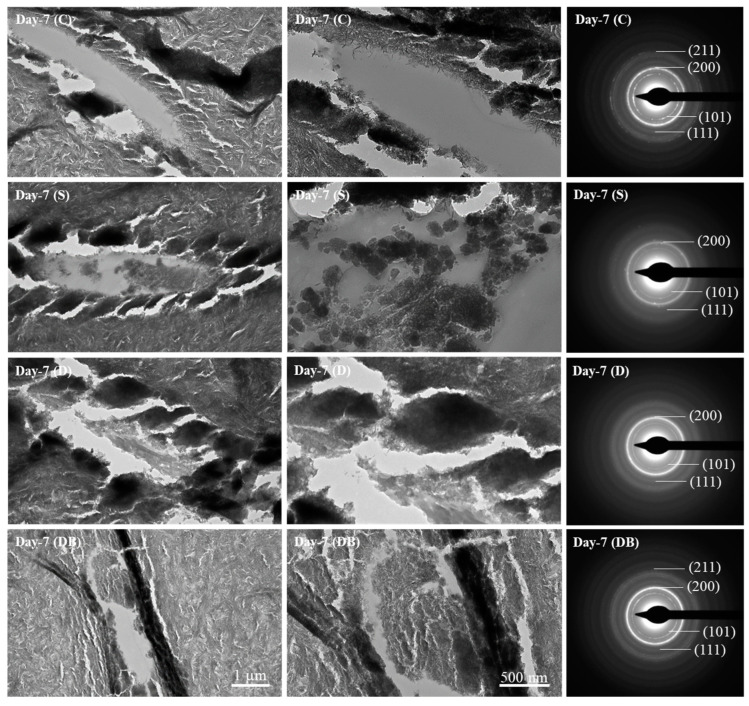
Bright-field TEM images (1 µm and 500 nm magnifications) of evaluated dentin groups. Group C = control; group S = Sensodyne Repair & Protect; group D = Dentalclean Daily Regenerating Gel; group DB = group D + Dentalclean regenerating booster. SAED analysis (third column) of selected deposition regions demonstrating the formation of hydroxyapatite.

Figure 4 demonstrates representative TEM images of treated dentin surfaces at the day 7 timepoint. Empty dentin tubules can be observed in the group C images. Dentin tubules in the images corresponding to groups S, D and DB disclose the presence of infiltrated precipitates. Bright-field TEM images and selected-area electron diffraction (SAED) patterns revealed the formation of nanocrystalline hydroxyapatite for all the treated samples. Each SAED pattern taken from the newly formed deposit (Figure 4, third column) exhibits polycrystalline-derived diffraction rings with lattice spacings of 0.281, 0.407, 0.526 and 0.388 nm, corresponding to the 211, 200, 101 and 111 reflections of hydroxyapatite [21]. The continuous circular pattern observed in the diffraction rings (i.e., (200) at day 7 (C)) implies a closely packed arrangement of crystallites compared with the non-completely continuous rings (i.e., (101) at day 7 (C)).

## 4. Discussion

In the present study, commercial dentifrices were applied on dentin surfaces for remineralization purposes. The SEM images (Figure 2) showed that all dentifrices achieved partial dentin tubule mineral coverage starting from the day 1 timepoint. A previous study has, however, reported a mineral layer formation on dentin only after 7 days of brushing evaluation using Dentalclean toothpaste [22]. Our results are in line with the previous report demonstrating formation of a remineralizing layer after brushing dentin with Dentalclean toothpaste associated with booster as soon as day 1 [13]. On the other hand, the group C sample images presented with more open tubules at all timepoints on SEM. Due to selective mineral removal caused by citric acid challenge, control images present with increased dentinal tubule lumen at day 14 [23]. Similarly, the dentin morphology of group C appears rougher at the day 14 evaluation in comparison to days 1 and 7. For the dentifrice-treated groups, AFM images (Figure 3) also depict surfaces that are rougher than the corresponding control images at the same timepoint, with visible distribution of circular deposits.

Comparable to a previous study [13], the EDX (Table 2, Table 3 and Table 4) elemental analysis along with the SEM and AFM images demonstrates that treatment with all dentifrices was able to modify the dentinal surface, also justifying the presence of elements such as Ca, F and P. These results lead to the rejection of the first proposed null hypothesis. Dentalclean dentifrice is regarded as a multifunctional phosphate-based dentifrice in an acidified and stabilized phosphate/fluoride complex. These compounds, in association with saliva and tooth structure, promote the formation of a mineralized layer (microporous; <2 nm) containing calcium, phosphate and fluorine in an acidic environment [23,24]. The additional calcium available in the booster (group DB) would favor formation of the mineralized layer, especially when applied on dentinal tissue that has less mineral content that enamel. At all timepoints, higher amounts of calcium (weight%) were indeed observed for group DB when compared with groups S and D.

Moreover, Sensodyne Repair & Protect is a bioglass-based dentifrice composed of calcium sodium phosphosilicate, bioactive glass and fluoride. With respect to their mode of action, bioglass particles interact with saliva, which results in an increase in local pH and the release of bioavailable calcium and phosphate ions, which, in turn, results in the formation of a mineralized layer on the dentin surface [25,26]. Hence, phosphate and calcium are precipitated from both the saliva and the dentifrice in alkaline pH, creating a calcium phosphate layer (mesoporous; 2 to 50 nm) over the tooth surface [24,27]. Elemental analysis also revealed relatively high amounts of Si (weight%) on dentifrice-treated groups at all timepoints (day 1: 6 to 12.7%; day 7: 4 to 7%; and day 14: 2.1 to 12.2%), compared with group C (day 1: 0.2%; day 7: 0.2%; and day 14: 0.7%) (Table 4). It has been previously reported that the silicon in dentifrices, such as that in Dentalclean, induces the formation of the calcium silicate responsible for enhancing hydroxyapatite nucleation and mineralization. Silicon induces the quick complexation of Ca and P, advancing the mineralization of the dentin surface by forming a Si-enriched hydroxyapatite mineral [15,22].

The day 1 Young’s modulus obtained for group C is comparable with sound dentin values reported in the literature [28]. In line with the SEM and AFM findings, a reduced mineral content due to citric acid challenge led to a significant decrease in the group C Young’s modulus at day 14 compared with days 1 and 7. The dentin surface on groups S and D presented significantly higher Young’s modulus values starting at day 7; the group S values stabilized from day 7 to day 14, whereas the group D values still significantly increased from day 7 to day 14. At day 14, the Young’s modulus of group D was statistically higher than group S, likely because the low pH favored the formation of combined calcium phosphate with silicon [24]. Although not statistically significant, compared with groups S and D, group DB had a slightly higher Young’s modulus value starting from day 1. This became statistically higher at day 7, with stabilized values at day 14. Interestingly, at day 7, the group DB Young’s modulus reached statistically similar values to groups D and DB at day 14, which are among the highest overall values. These results suggest that although the addition of the booster in group DB may not have increased mineral formation in comparison with group D, it might have accelerated the process of mineral deposition. The Young’s modulus values of groups D and DB at day 14 (Table 5) are higher than the previously reported Young’s modulus values obtained for remineralized dentin [29]. According to the SEM and AFM images, as well as the Young’s modulus results, the acidic challenge (day 14) was not able to remove the mineralized layer formed on dentin by the dentifrices, nor reduce the Young’s modulus, which leads to rejection of the second null hypothesis.

TEM imaging (Figure 4) complemented the SEM and AFM results by showing deposited hydroxyapatite inside the dentin tubules at day 7. A previous report has also demonstrated the formation of mineralized areas inside dentinal tubules after 5 daily applications of Dentalclean associated with booster [13]. All reflections from the (101), (111), (200) and (211) planes can be identified as polycrystalline hydroxyapatite after SAED indexing [21,30]. SAED characterization has also been previously performed to disclose the local crystallinity of dentin apatite crystals underneath a hybrid layer before and after an acid–base challenge [31]. Likewise, SAED analysis revealed the formation of characteristic crystallographic planes for hydroxyapatite inside dentin tubules after brushing with a calcium silicate and sodium-phosphate-containing toothpaste [32]. The TEM observations are consistent with previous studies showing that remineralizing dentifrices are effective in controlling teeth hypersensitivity. In fact, a previous clinical study showed that Sensodyne Repair & Protect was able to reduce tooth sensitivity after single use in 90% of 48 patients tested [33]. Dentalclean dentifrice has also been reported to significantly reduce dentin hypersensitivity, evaluated by means of pain score, immediately after initial use, as well as after 1 week of clinical use by 53 patients. The mineral formation caused by these dentifrices gradually covers dentin and penetrates dentinal tubules, decreasing dentinal fluid flow and thus minimize the dentin hypersensitivity [15,16]. The abovementioned clinical outcomes observed with the use of both Sensodyne Repair & Protect and Dentalclean dentifrices align with the findings of this study. It is important to note that in the present study, toothbrushing and surface characterization were conducted on freshly extracted adult molar teeth and that the current results are applicable to sound dentin. To unravel the remineralizing properties of the evaluated toothpastes, long-term clinical trials and epidemiological studies should be considered in future studies. Most importantly, future clinical trials should be conducted with relevance to populations under high risk for tooth demineralization such as orthodontic and pediatric patients, patients at high risk for caries or those diagnosed with molar incisor hypomineralization.

## 5. Conclusions

Chemical and ultrastructural results showed that the use of the studied dentifrices let to mineral formation that gradually covered dentin samples as well as promoted penetration into dentinal tubules. The citric acid challenge was not able to prevent formation nor remove the newly formed mineralized layer.

## Figures and Tables

**Table 1:** Description of the product composition, product manufacturers and application protocols.

Group	Toothpaste	Composition	Manufacturer	Protocol of Application
C	Control (artificial saliva)	Carboxymethylcellulose, 50 g; methyl 4-hydroxybenzoate, 10 g; MgCl_2_H_2_O, 0.2944 g; CaCl_2_·2H_2_O, 0.830 g; K_2_HPO_4_, 1.462 g; KCl, 3.124 g; for 1 L preparation.	-	Specimens remained immersed (1 mL/specimen) during fourteen consecutive days
S	Sensodyne Repair & Protect	Calcium sodium phosphosilicate (NOVAMIN) 5% *w*/*w*, sodium fluoride (0.2299% *w*/*w*) (fluoride 0.104% *w*/*w*)	GlaxoSmithKline, Mississauga, ON, Canada	Manually brushed for two minutes, two times/day, during fourteen consecutive days
D	Dentalclean Daily Regenerating Gel	1450 ppm sodium fluoride, glycerin, silica, sorbitol, sodium lauryl sulfate, aqua, aroma, PEG (polyethylene glycol)-12, cellulose gum, O-phosphoric acid, xylitol, tetrasodium pyrophosphate, sodium saccharin, triclosan, menthol, mica, sodium benzoate	Dentalclean, Londrina, PR, Brazil	Manually brushed for two minutes, two times/day, during fourteen consecutive days
B	Dentalclean Regenerating Booster	5% Calmix (calcium carbonate, tricalcium phosphate, silica, glycerin, triclosan, lauryl, saccharine, water)	Dentalclean, Londrina, PR, Brazil	

**Table 2:** Mean percentage values of elemental mapping (W = weight; A = atomic) obtained for day 1.

Element	C		S		D		DB	
	W%	A%	W%	A%	W%	A%	W%	A%
K L	46	43	23	20	23	18	40	35
C K	6.1	21	3.0	8.0	3.0	9.0	5.0	11
Ca L	18	18	9.0	10	13	10	15	12
N K	2.6	6.2	3.0	7.0	3.0	7.0	4.0	8.0
O K	4.3	9.5	16	32	16	32	12	21
F K	0.1	0.2	0.1	0.2	0.2	0.4	0.1	0.2
Na K	0.3	0.5	0.4	0.7	0.6	0.8	0.4	0.5
Mg K	0.3	0.6	0.7	0.9	0.5	0.7	0.5	0.6
Al K	0.3	0.5	0.7	0.9	0.6	0.7	0.4	0.4
Si K	0.2	0.2	12.7	15	10	12	6.0	6.0
P K	0.9	0.3	0.5	0.6	0.4	0.4	0.3	0.3
Zr L	1.3	0.5	4.0	1.5	1.5	0.5	3.0	1.0
Cl K	1.0	1.0	0.7	0.7	0.9	0.8	0.8	0.7

C: control (artificial saliva; no toothpaste); S: Sensodyne Repair & Protect; D: Dentalclean Daily Regenerating Gel; and DB: D group + Dentalclean regenerating booster.

**Table 3:** Mean percentage values of elemental mapping (W = weight; A = atomic) obtained for day 7.

Element	C		S		D		DB	
	W%	A%	W%	A%	W%	A%	W%	A%
K L	57	47	20	5.0	13	10	32	28
C K	6	16	1.0	11	2.0	6.0	4.0	11
Ca L	17	13	13	4.0	12	9.0	18	15
N K	5	11	2.0	42	2	6.0	4.0	10
O K	4	8.0	21	0.8	23	44	11	23
F K	0.1	0.1	0.5	1.0	0.5	0.8	0.3	0.4
Na K	0.3	0.5	0.8	1.0	1.0	1.0	0.6	0.9
Mg K	0.3	0.4	0.9	0.8	0.8	1.0	0.6	0.8
Al K	0.2	0.3	0.7	4.0	0.9	1.0	0.6	0.8
Si K	0.2	0.3	4.0	7.0	7.0	7.0	5.0	6.0
P K	0.2	0	7.0	0.7	7.0	7.0	0	0.0
Zr L	1.3	1.0	2.0	4.0	6.0	2.0	23	4.0
Cl K	0.7	0.6	0.8	5.0	0.6	0.6	32	28

C: control (artificial saliva; no toothpaste); S: Sensodyne Repair & Protect; D: Dentalclean Daily Regenerating Gel; and DB: D group + Dentalclean regenerating booster.

**Table 4:** Mean percentage values of elemental mapping (W = weight; A = atomic) obtained for day 14.

Element	C		S		D		DB	
	W%	A%	W%	A%	W%	A%	W%	A%
K L	41.5	35.6	30.1	27.0	17.9	15.8	27.4	23.1
C K	6.2	17.0	1.1	3.1	1.1	3.2	5.1	13.4
Ca L	19.2	16.0	12.5	10.9	7.9	6.8	14.5	11.6
N K	3.9	9.3	2.0	4.9	2.1	5.0	4.4	10.2
O K	6.1	12.8	15.5	33.9	20.2	42.2	12.5	24.8
F K	0.1	0.2	0.3	0.6	0.3	0.6	0.2	0.3
Na K	0.6	0.9	0.6	0.9	0.6	0.9	0.6	0.8
Mg K	0.5	0.7	0.8	1.1	0.7	0.9	0.4	0.6
Al K	0.5	0.6	0.7	0.9	0.6	0.8	0.6	0.8
Si K	0.7	0.9	2.1	2.7	12.2	14.4	7.6	8.6
P K	0.3	0.2	5.3	6.0	2.6	2.9	0.3	0.3
Zr L	9.3	3.4	5.7	2.3	3.1	1.2	10.1	3.6
Cl K	0.9	0.9	0.6	0.6	0.8	0.8	0.8	0.7

C: control (artificial saliva; no toothpaste); S: Sensodyne Repair & Protect; D: Dentalclean Daily Regenerating Gel; and DB: D group + Dentalclean regenerating booster.

**Table 5:** Mean values (s.d.) for dentin Young’s modulus (GPa).

Property		Groups	C	S	D	DB
	Timepoint					
Young’s Modulus	Day 1		18.4 (0.14) ^Aa^	18.5 (0.30) ^Aa^	18.8 (0.39) ^Aa^	19.5 (0.78) ^Aa^
	Day 7		16.9 (0.77) ^Aa^	23.3 (1.24) ^Bb^	24.4 (1.96) ^Bb^	36.3 (0.54) ^Cb^
	Day 14		14.6 (2.68) ^Ab^	25.3 (2.24) ^Ab^	35.3 (0.66) ^Bc^	37.7 (1.13) ^Bb^

Identical upper-case letters indicate no significant differences among lines. Identical lower-case letters indicate no significant differences within each column.

## Data Availability

Due to intellectual property issues, data will be made available upon request.
